# Longitudinal cortical atrophy in cases of AT concordance and discordance

**DOI:** 10.1002/alz.093826

**Published:** 2025-01-09

**Authors:** Konstantinos Ioannou, Rosaleena Mohanty, Konstantinos Poulakis, Antoine Leuzy, J‐Sebastian Muehlboeck, Elena Rodriguez‐Vieitez, Eric Westman, Konstantinos Chiotis

**Affiliations:** ^1^ Department of Neurobiology, Care Sciences and Society, Center for Alzheimer Research, Karolinska Institutet, Stockholm Sweden; ^2^ Division of Clinical Geriatrics, Center for Alzheimer Research, Department of Neurobiology, Care Sciences and Society, Karolinska Institutet, Stockholm Sweden; ^3^ Wallenberg Centre for Molecular and Translational Medicine, Sahlgrenska Academy, University of Gothenburg, Gothenburg, Västergötland Sweden; ^4^ Department of Neurology, Karolinska University Hospital, Stockholm Sweden

## Abstract

**Background:**

Although ß‐amyloid and tau PET positivity (A+T+) has been related to neurodegeneration and cognitive decline in Alzheimer’s disease (AD), the driving force of neurodegeneration in discordant AT cases remains controversial. We investigated the impact of AT status on longitudinal rates of cortical atrophy and cognitive decline.

**Method:**

A subset of 349 individuals (cognitively unimpaired [CU; n = 230], cognitively impaired [CI; n = 119]) with ß‐amyloid and tau PET (a priori baseline), longitudinal MRI (interval; Mean = 4.5±3.3 years, scans/individual; Median = 3, IQR = [2, 8]), and a cognitive follow‐up = 2 years, was selected from the ADNI dataset. We classified the individuals based on their AT PET status (A+; global ß‐amyloid = 20 CL, T+; composite temporal region = 1.34 SUVR) and clustered them based on their annual rates of cognitive decline on the ADAS‐Cog13 (fast vs. slow decliners [FDs vs. SDs]). Baseline AD‐signature cortical thickness was compared across AT groups. We employed a linear mixed‐effects model to assess the interaction between AT status and longitudinal cortical thickness across all cortical regions.

**Results:**

Demographic characteristics were similar across AT groups (A‐T‐; n = 192, A‐T+; n = 5, A+T‐; n = 106, A+T+; n = 46). Most A+T+ individuals (70%) were CI, whereas most A+T‐ (71%), A‐T+ (80%), and A‐T‐ (71%) individuals were CU. Similarly, most A+T+ individuals (85%) were characterized as FDs, whereas most A+T‐ (74%), A‐T+ (60%), and A‐T‐ (88%) individuals were characterized as SDs. A+T+ individuals showed significantly lower AD‐signature cortical thickness (MeanA+T+ = 2.78±0.19 mm) compared to A+T‐ (MeanA+T‐ = 2.89±0.15 mm; P = 0.0023) and A‐T‐ (MeanA‐T‐ = 2.89±0.15 mm; P<0.001) individuals, and the highest Aß burden (Mean = 85.3±30.6 CL; P<0.0001) at baseline. AT status significantly influenced longitudinal cortical thickness. A+T+ individuals showed significantly steeper cortical thinning rates compared to A‐T‐ individuals, primarily in temporoparietal areas. Despite similar group‐level cortical thinning rates with A‐T‐ individuals, A+T‐ individuals showed heterogeneous patterns of cortical atrophy at the individual level. These findings remained consistent even when conducting separate analyses for CU and CI individuals.

**Conclusion:**

Unlike AT discordance, concordant AT positivity exhibited homogeneous cortical atrophy patterns consistent with AD. Considering the concurrent high prevalence of mixed pathologies in neuropathological AD, we further aim to explore the underlying neurodegenerative force in discordant AT cases.

